# Does type of provider matter for staff well-being? a cross-sectional study of residential care home workers’ job demands and resources

**DOI:** 10.1177/10519815241300294

**Published:** 2025-01-21

**Authors:** Tomas Lindmark, Sven Trygged, Maria Engström

**Affiliations:** 1 Department of Social Work, Faculty of Health and Occupational Studies, University of Gävle, Gävle, Sweden; 2 Department of Caring Science, Faculty of Health and Occupational Studies, University of Gävle, Gävle, Sweden; 3 Medicine College, Lishui University, Lishui, China

**Keywords:** assistant nurses, burnout, psychological, job satisfaction, nursing homes, ownership, personnel turnover, psychological well-Being, working conditions

## Abstract

**Background:** Marketisation trends have introduced new elements in residential care homes, potentially related to the psychosocial work environment and well-being of care workers. **Objective:** This study examined differences in job demands and resources across public, outsourced, and private residential care home providers and their associations with care workers’ burnout, job satisfaction, and turnover intentions. **Methods:** Data from 253 care workers across 19 residential care homes in three municipalities were analysed using a cross-sectional design, with a 45.3% response rate. We applied the Job Demands-Resources theory and the Copenhagen psychosocial questionnaire, conducting analyses of variance and multiple regressions with Generalised Estimating Equations to account for nested data. **Results:** Outsourced care workers reported higher emotional demands than those in the public sector, while private providers offered greater influence and supervisor support compared to public ones. Burnout levels were significantly higher in the medium-sized municipality compared to the small one, while provider type was not significant. Private care workers reported higher job satisfaction, but public sector workers reported better work-life balance. Approximately 60% of respondents considered leaving their jobs at least occasionally, with public sector workers reporting higher turnover intentions than those in for-profit settings. **Conclusions:** The study highlights the need for targeted work environment improvements, including better leadership and support in the public sector, addressing emotional demands in outsourced settings, and encouraging full-time employment to support work-life balance in the private sector. Stakeholders should prioritise improving job resources to improve care workers’ well-being, especially amid budget constraints and profit goals.

## Introduction

The ongoing marketisation of care has introduced new layers of complexity in long-term residential care homes for older adults, including variations in providers, such as for-profit and nonprofit entities. This marketisation trend, characterised by the introduction of market mechanisms, private for-profit providers, and a focus on consumer choice,^1^ may increase pre-existing challenges in eldercare. Challenges reported are, for example, labour shortages,^2^ high job demands and high turnover intentions,^3^ and substantial stress impacting care workers’ mental and physical health.^4–6^ From 2002 to 2020, Swedish municipalities faced consistent budget cuts in eldercare, which intensified in 2020, where nearly all municipalities imposed stricter budgetary constraints.^7^ Financial constraints may increase workloads, reduce resources, and potentially lead to job cuts for care workers.^8^ These issues might be particularly pronounced in for-profit settings, where some providers focus on maximising profits at the expense of staffing levels.^9^

Studies show mixed results regarding links between provider types and staff outcomes in residential care homes. Lindmark et al.'s^10^ systematic review found inconsistent results, but the results regarding staff well-being generally favoured nonprofit settings. Studies have also indicated lower staffing levels in for-profit care^11,12^ and less favourable work environments when public organisations are outsourced.^13^ Furthermore, the procurement documents tied to outsourcing increasingly call for empowering the residents, often resulting in additional demands on the care workers without increasing job resources.^14^ This trend is substantiated by lower turnover intentions in nonprofit residential care homes,^15^ consistent with the argument that for-profit homes often compromise on staffing levels, affecting the work environment and increasing turnover intentions.^9^

While previous studies have explored care quality under different provider models in Swedish residential care homes,^12,16^ less attention has been given to the association between provider type and care workers’ well-being, and existing research has shown mixed results.^10^ This study aimed to fill this gap by investigating differences in the psychosocial work environment and well-being between provider types (public, outsourced for-profit, and private for-profit residential care homes) and associations between job demands, job resources, provider types and care workers’ burnout, job satisfaction, and turnover intentions across three municipalities.

### Background

In Sweden, as in other Nordic countries, eldercare is grounded in a social care model where services are publicly funded, ensuring universal access regardless of income and emphasising equality and broad coverage.^1^ This model is a core component of the Nordic welfare state, characterised by extensive public responsibility for social services financed primarily through general taxation. Municipalities are central to this system, responsible for delivering and overseeing eldercare, ensuring that all citizens receive high-quality services, irrespective of their financial situation.^1,17^

Since the 1990s, there has been a shift towards incorporating market principles prompted by economic recession, New Public Management (NPM) ideas, and new legislation. The marketisation of care emphasises for-profit providers and customer choice models, which are proposed to boost care users’ autonomy. Legislative shifts, such as the Local Government Act of 1991 and the Act on Public Procurement, introduced 1992 and updated in 2016, have allowed municipalities to outsource eldercare services to private providers through a competitive bidding process. Meanwhile, the Act on the System of Choice in the Public Sector, implemented in 2009, support a system where care users select their preferred service provider. Not all municipalities have implemented the three legislative acts above, but most municipalities have seen changes based on NPM ideals.^18^ This shift has notably increased the presence of for-profit care providers, which now form a substantial portion of the eldercare sector, raising questions about care standards and fairness.^17,19,20^

Swedish residential care homes are operated by three main types of providers: public, private (including both for-profit and nonprofit), and outsourced providers. Regardless of the provider type, residential care is primarily funded by municipalities, ensuring a consistent financial foundation.^17^ Public providers, managed by municipalities, are associated with nonprofit status.^19^ Outsourcing involves municipalities contracting private providers, usually large for-profit care chains,^20^ to manage publicly owned facilities through a procurement process, typically under the Act on Public Procurement.^14,19^ In these cases, the municipality retains ownership, but the private provider handles daily operations. Private for-profit providers, however, generally own and operate their facilities independently, distributing any surplus income to shareholders or owners.^19^ These providers often operate within a customer choice model, supported by legislation like the Act on System of Choice.

This market transformation has increased the percentage of private care homes to around 20%,^21^ where around 3% are nonprofit private providers and the remaining 17% are for-profit. When facilities are privately operated, terminating contracts for subpar services becomes a challenge for municipalities. Such termination can disrupt the lives of existing residents.^17^ Despite NPM principles highlighting market efficiencies, these changes have sparked debates over their effects on the core values of fairness and the welfare ethos that defines Swedish eldercare.^22^ Specifically, the move towards marketisation raises concerns about the principles of universalism, inclusiveness, and equality that the Nordic welfare model stands on. The rise of for-profit providers and the focus on consumer choice suggest a shift towards a more individualised, market-led approach.^17^ Despite the anticipated benefits of marketisation, such as cost-efficiency and consumer choice, criticisms have emerged regarding its potential to compromise care quality and worker conditions.^20,22,23^ Håkansson^24^ found that stringent financial controls in Swedish eldercare often prioritise market-driven principles, such as metrics and reporting, over promoting trust and autonomy for care workers in their roles.

### The provider's role in shaping care quality and working conditions

Armstrong et al.^23^ highlight that for-profit providers’ cost-cutting measures in eldercare often include prioritising part-time staff over full-time positions. This reliance on part-time workers may disrupt the development of stable relationships between care workers and users. Research shows that care quality varies between nonprofit and for-profit care homes, with the latter often having lower staffing levels.^9,11,25,26^ Bos et al.^9^ indicated that for-profit residential care homes in the United States may prioritise financial performance, possibly affecting care worker well-being. Lindmark et al.'s systematic review^10^ summarised how profit incentives relate to care workers’ psychosocial work environment and well-being. The findings were generally mixed but indicated slightly better well-being outcomes in nonprofit settings compared to for-profit ones, for example regarding turnover intentions, stress, and burnout. The results concerning job satisfaction and type of provider were mixed across various studies. A recent systematic review found no clear relationship between facility ownership and job satisfaction among registered and licensed practical nurses.^27^

In Sweden, existing research has predominantly focused on care quality rather than the work environment, with mixed results.^12,16,26^ Feltenius^19^ argues that there are no significant differences between providers from a service provision perspective due to the stringent municipal governance standards across public and private entities. However, similar service provision does not mean that the work environment is the same. In terms of working conditions, private Swedish residential care homes tend to employ more workers on zero-hour contracts.^28^ The public sector employs a higher proportion of assistant nurses (47%) compared to private providers (38%).^29^ Few studies have explicitly examined the type of provider and its association with the staff's work environment. One study found that employees in cooperatives experienced more participation than those in other provider types.^30^ Simmons et al.^31^ indicated that poorer working conditions in the long-term care sector in Sweden compared to Austria could be due to Sweden's higher proportion of for-profit providers.

### Theoretical framework

Our study utilises the Job Demands-Resources (JD-R) theory to investigate psychosocial work environments across care home providers. The theory distinguishes between job demands, which can strain employees, and job resources, which support employees and buffer job strain.^32^ JD-R outlines two fundamental mechanisms: the health impairment process, which increases strain and the risk of burnout and other health issues, and the motivational process, which enhances positive outcomes like job satisfaction and commitment. These processes, in turn, may affect job performance.^32^

Prior research in residential care homes using JD-R theory has consistently shown the significance of job demands and resources in affecting turnover intentions, work-related stress, job performance, and burnout.^33–35^ We extend this research by examining JD-R's applicability in varied organisational contexts, as suggested by Bakker and Demerouti.^32^

We use the Copenhagen Psychosocial Questionnaire (COPSOQ) to assess job demands and resources, which aligns well with the JD-R theory.^36^ Our study explores leadership as a job resource, which is noted as an area for further research.^32^ We also incorporate social capital as a job resource, in line with the COPSOQ framework^37^ and supported by previous research.^38–40^ Furthermore, supportive social networks are a crucial factor in reducing turnover among assistant nurses and improving work attendance.^41^

In this study, we examine burnout as an outcome indicative of the health impairment process, while job satisfaction represents the motivational process. We also look at turnover intention, which could indicate either of the processes depending on the result. Highlighting the relevance of these processes, Szebehely et al.^3^ found high turnover intentions among care workers. Lindmark et al.^10^ found stress and burnout to be more prevalent in for-profit settings than in nonprofit ones, with studies on job satisfaction and turnover intentions showing varied results. Additionally, this study explores possible aspects to include in the JD-R theory, as recommended by Bakker and Demerouti.^32^ These aspects include the potential buffering effect of job resources against job demands and the application of the JD-R theory across varied organisational settings, specifically within public, outsourced, and private care homes. We also consider the multilevel perspective, which highlights how organisational-level factors, such as leadership practices and decision-making processes, can trickle down to affect team dynamics and individual outcomes.^42^ We include leadership and social capital as job resources that may influence the psychosocial work environment in these different contexts. By examining these factors across different provider types, our study seeks to understand variations in organisational structures.

### Aim, research questions and hypotheses

**Figure 1.**
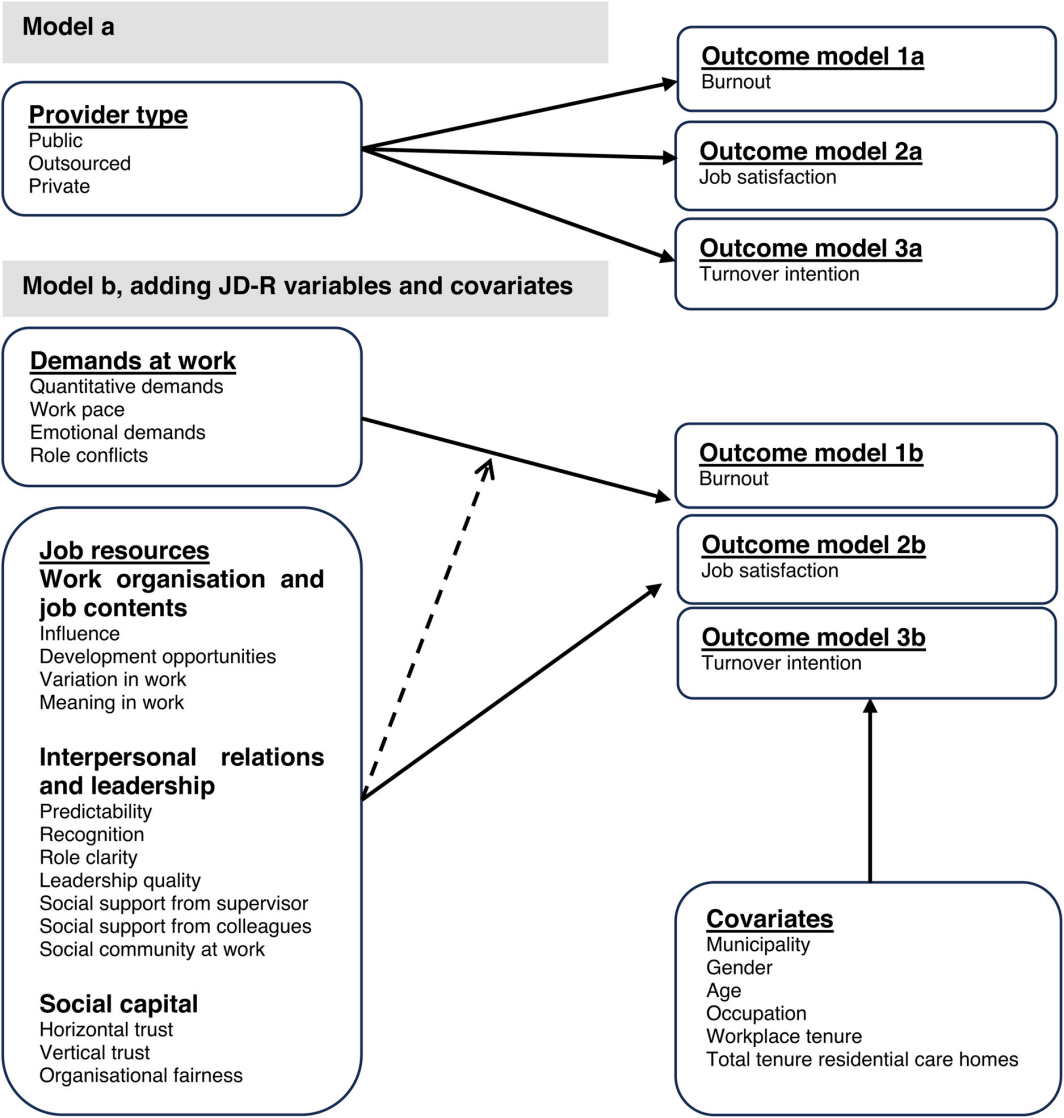
Inspired by the job demands-resources theory, the conceptual model illustrates the proposed associations between provider type, job demands, job resources, and each outcome. As we had three outcomes, we ran three models: 1 burnout, 2 job satisfaction, and 3 turnover intentions. Models 1–3a examine the association between provider types and outcomes. Models 1–3b build upon model a and add variables: job demands and resources. Model 1–3b also includes covariates and explores the moderating role of job resources (dotted lines) on the relationship between job demands and outcomes.

To sum up, the ongoing marketisation of eldercare, marked by the rise of for-profit providers and challenges like labour shortages, high demands, and turnover, raises concerns about care workers’ well-being. Against this backdrop, and considering mixed results regarding the influence of different care providers on staff well-being,^10^ this study aimed to examine a) differences in job demands and resources across public, outsourced for-profit, and private for-profit residential care home providers. Additionally, b) associations between job demands, job resources, provider type, and the outcomes of burnout, job satisfaction, and turnover intentions among care workers. We specifically compare three types of providers: outsourced for-profit, private for-profit, and public nonprofit. For a comprehensive overview of the variables analysed for the second part of the aim and their interrelationships, see Figure 1.

Given the mixed results in previous studies regarding associations between provider types and care worker outcomes,^10–13^ we formulated two exploratory research questions (Q1 and Q2). Our study focuses on burnout, job satisfaction, and turnover intentions among care workers. While these variables are well-documented in eldercare, their association with provider type is underexplored. Previous research, primarily from North America, has shown mixed results regarding turnover intentions and job satisfaction across different care providers,^10^ with stress and burnout generally lower in nonprofit settings. This study adds to the research by investigating how market-driven changes across different provider types are associated with care workers’ well-being within the Nordic social care model, characterised by a strong welfare state and publicly funded services.^1^ The findings may be relevant not only for Sweden but also for other countries with similar welfare systems.

**Q1:** Are there differences in job demands and resources across provider types (public, outsourced for-profit, and private for-profit)?

**Q2:** Are there associations between the type of residential care home provider (public, outsourced for-profit, and private for-profit) and care workers’ burnout, job satisfaction, and turnover intentions?

For aspects linked to the JD-R theory, supported by previous research,^32–35^ we posit two hypotheses (H1 and H2). H1 aligns with the theory's premise that high job demands, without adequate resources, lead to burnout and reduced job satisfaction. These factors may increase turnover intentions as well. H2 builds on Bakker and Demerouti's^32^ suggestion to investigate how job resources may buffer the adverse effects of job demands.

**H1:** Job demands are associated with increased burnout, turnover intentions, and lower job satisfaction, whereas job resources are associated with lower burnout and turnover intentions but higher job satisfaction.

**H2:** Job resources moderate the association between job demands and burnout, job satisfaction, and turnover intention. Higher job resources may buffer the negative impact of job demands on the mentioned outcomes.

## Methods

### Methodological approach

The study had a cross-sectional comparative and correlational design. Initially, the intent was to compare nonprofit and for-profit sectors, grouping private and outsourced providers together. However, based on recent findings,^26,43^ we differentiated for-profit providers into private and outsourced settings, as different types of private providers – particularly those influenced by private equity – show distinct practices and priorities, which may influence the psychosocial work environment.

### Setting and participants

In Sweden, residential care homes are facilities that provide 24-h care to older adults in need of continuous health and social support services. The care work includes various tasks, including handling medication administration, rehabilitation, social activities, documentation and cleaning and cooking.^44,45^ The workforce in Swedish eldercare is highly gendered, with around 89% being women^46^ and approximately one-third coming from non-European countries.^47^

Included were care workers employed in public, outsourced, and private residential care homes in three Swedish municipalities, categorised as small, medium-sized, and large. We aimed to secure a nearly equal number of participants from public and private care homes to achieve a balanced representation. Nineteen of the 29 contacted residential care homes agreed to participate: 9 publicly owned, 5 outsourced to private providers, and 5 entirely privately owned and operated. All care workers at these homes were invited to participate (Table 1).

**Table 1.** Background information concerning the participants based on provider type.

Variable	Public (n=136)	Outsourced (n=42)	Private (n=75)	Overall (n=253)
Sex				
Male	18 (13%)	2 (5%)	7 (9%)	27 (11%)
Female	117 (86%)	40 (95%)	64 (85%)	221 (87%)
Age	Range: 20–65	Range: 25–69	Range: 19–68	Range: 19–69
	Mean: 48.1	Mean: 49.5	Mean: 43.0	Mean: 46.9
	SD: 10.8	SD: 10.6	SD: 12.4	SD: 11.5
Workplace tenure	Range: 0–30	Range: 0–25	Range: 0–12	Range: 0–30
	Mean: 7.1	Mean: 8.9	Mean: 4.8	Mean: 6.7
	SD: 6.2	SD: 8.4	SD: 2.9	SD: 6.1
Total tenure residential care homes	Range: 0–45	Range: 0–41	Range: 1–37	Range: 0–45
	Mean: 17.4	Mean: 20.9	Mean: 13.9	Mean: 17.0
	SD: 11.8	SD: 12.4	SD: 10.7	SD: 11.8
Municipality				
Small	33 (24%)	17 (41%)	0	50 (20%)
Medium-sized	74 (55%)	25 (59%)	33 (44%)	132 (52%)
Large	29 (21%)	0	42 (56%)	71 (28%)
Occupation				
Care aide	26 (19%)	5 (12%)	14 (19%)	45 (18%)
Assistant nurse	106 (78%)	33 (79%)	46 (61%)	185 (73%)
Registered nurse	3 (2%)	3 (7%)	9 (12%)	15 (6%)
Other	1 (1%)	1 (2%)	4 (5%)	6 (2%)
Employment type				
Full-time	85 (62%)	11 (26%)	34 (45%)	130 (51%)
Part-time	38 (28%)	27 (64%)	37 (49%)	102 (40%)
Education level				
Elementary school or equivalent	17 (12%)	3 (7%)	5 (6%)	25 (10%)
High school or equivalent	107 (79%)	33 (79%)	44 (59%)	184 (73%)
University or equivalent	11 (8%)	6 (14%)	23 (31%)	40 (16%)
Type of education *				

*See Supplementary Materials.

Care workers in residential care homes in Sweden often hold the title ‘undersköterska’, which we refer to as ‘assistant nurses’ in this study. Swedish assistant nurses typically complete a two-to-three-year education.^3^ The sample also included ‘care aides’ (‘vårdbiträden’ in Swedish), who may possess varying levels of formal education, from specific healthcare training to no formal healthcare qualifications. Staff with higher education, including registered nurses and a few other groups, such as physiotherapists, were also included in the sample.

Our regression models included 8 to 11 variables. Following the guideline of ten participants per variable, the sample size was determined to ensure sufficient power to detect an effect size of approximately 0.40, as recommended by Cohen.^48^ In total, 558 full-time or part-time care workers engaged in hands-on care were invited to participate. The criteria excluded staff not directly involved in residents’ health and social care, such as managers and janitors. We also excluded zero-hour contract workers from the study as they rotate across various units, which complicates responses to unit-specific survey questions.

### Materials

The Copenhagen Psychosocial Questionnaire (COPSOQ III) measures the organisational and social work environment. It is suitable for use in varied workplaces and has been validated in several languages and countries.^49,50^ For examples in English of items included in the questionnaire, see Burr et al.^50^ It encompasses six domains: Demands at Work, Work Organisation and Job Contents, Interpersonal Relations and Leadership, Work–Individual Interface, Social Capital, and Health and Well-being. We utilised 63 of the 69 questions in the Swedish version of COPSOQ III,^50^ omitting six questions (concerning worries about unemployment and technological replacement) that fell outside our research scope.

Given the extensive scope of job demands and resources covered by COPSOQ, we opted for a more consolidated approach due to concerns about our sample size and overfitting. Instead of employing all dimension scales (4 job demands and 14 job resources), we aggregated them and used the broader domains in the multiple regressions. Previous research has tested and used all domains across dimensions^51^ and specific domains.^52,53^ We used the domain ‘Demands at Work’ to measure job demands. Although COPSOQ III categorises the dimension ‘Role Conflicts’ under the domain ‘Interpersonal Relations and Leadership’, we included it in the ‘Demands at Work’ domain. This adjustment was made to reflect the conventional view of role conflicts as a job demand rather than a resource.^32,42^ We used the three existing domains for job resources: ‘Work Organisation and Job Contents’, ‘Interpersonal Relations and Leadership’, and ‘Social Capital’.

Burnout was measured as a dimension, while turnover intention was assessed with a single-item question: ‘How often do you consider looking for work elsewhere?’ The response was reversed from the COPSOQ guidelines, with higher values indicating higher turnover intentions. Job satisfaction was also measured as a single item: ‘Regarding your work in general, how pleased are you with your job as a whole, everything taken into consideration?’ While respondents initially answered on a five-point scale, including options such as ‘often’ or ‘rarely’, their responses were converted to a 0–100 scale for analysis, in line with COPSOQ guidelines.^50^ After the COPSOQ questions, there were also eleven demographic questions regarding, for example, gender, age, workplace tenure, and total tenure in residential care homes.

### Data collection

The data collection took place between January and November 2022. Firstly, managers at the residential care homes were contacted. Upon receiving approval from first-line managers, arrangements were made to attend regular workplace meetings and distribute the questionnaire there. The first author attended multiple meetings at each workplace for study information, two to three meetings at smaller residential care homes and up to six meetings at larger facilities. The questionnaire could also be accessed through a computer or mobile phone using a QR code on the first page of the survey. Electronic mailing lists were unavailable for some workplaces, and a few staff members were absent during the workplace meetings. In these cases, surveys were provided to first-line managers, who distributed them to the missing staff members. Participants received an envelope containing an informed consent document and the questionnaire with a unique identifying code. A securely locked post box was placed at each workplace for three weeks to collect completed paper questionnaires. Some workplaces allowed the care workers to complete the survey during working hours, while others did not.

### Data analysis

The data were analysed using IBM SPSS version 27. The internal reliability of the dimensions was checked using Cronbach's alpha, with values ranging from .77 to .91. For partially answered scales within the COPSOQ, we followed Berthelsen et al.^49^ and included respondents who completed at least half of the scale items. The highest proportion of missing responses for any COPSOQ variable was 1.2%. The significance level was set to p ≤ .05 for analyses of variance (ANOVA) and Generalised Estimating Equations (GEE) analyses.

We conducted ANOVA tests to investigate differences in JD-R variables between public, outsourced, and private providers (**Q1**). Our comparative analyses examined both domain-level and dimension-level variables. Given the potential for unequal group sizes, we employed Tukey's Honest Significant Difference (HSD) test as a post hoc method to adjust for multiple comparisons and identify specific group differences where the ANOVA indicated significant effects.^54^ We followed the general benchmarks^48^ for evaluating eta-squared effect sizes, classifying them as small (≥.01), medium (≥.06), and large (≥.14).

Regarding the GEE models, we first looked at the independent JD-R variables’ correlation with each of the three outcomes using bivariate correlations. All JD-R variables were significant for all outcomes. We also checked the correlation of demographic variables with the outcomes using Pearson's R and t-test for gender (see Supplementary Materials). Variables with p-values of .10 or less were included in the GEE analyses. Municipality and type of occupation were included as control variables for all outcomes to address potential biases. We controlled for municipality due to the high local autonomy in Swedish municipalities, which leads to variations between municipalities concerning the structure of long-term residential care.^18,55^ Occupation was also controlled due to the notably low representation of registered nurses, particularly among public providers. We assessed multicollinearity among predictor variables by computing Variance Inflation Factors (VIF) for the residuals. The VIF values were as follows: job demands (1.1), interpersonal relations and leadership (2.9), work organisation and job content (1.5), and social capital (2.4) for all outcomes. None of these values exceeded the widely accepted cutoffs of 3.3 or 5.^56^

Following Huang's^57^ suggestion that GEE is suitable for cross-sectional clustered data, we chose GEE over Multilevel Modelling for multiple regressions. In the GEE models, care workers were grouped across 19 distinct residential care homes in three different municipalities and across three different provider types. We used an independent correlation structure for all outcomes because it had the lowest QIC value, that is, the best goodness of fit. GEE makes fewer assumptions regarding the distribution of residuals and correlation structures than Multilevel Modelling,^57^ offering robustness, particularly given the small sample size. We assessed the residuals of each GEE model using histograms, box plots, and Q-Q plots, which revealed no serious deviations from normality.

At first, in the GEE models, we included only the type of provider (public sector as the reference group) in the outcomes to see the initial regression effect of provider type (Models 1–3a, **Q2**). In the second step, we included the JD-R and demographic variables from the bivariate correlations with p-values ≤0.1 (Models 1–3b, **Q2**, **H1**). Interaction effects, in the form of the buffering effect of job resources on job demands (**H2**), were checked in line with Bakker and Demerouti's^32^ recommendations. If significant, the interaction was kept in the final model.

## Results

Of the initial 558 staff members, 485 received the questionnaire (73 were on vacation, on parental or sick leave, or said no), and 253 completed it. This resulted in a response rate of 52.2% among those who received the questionnaire and 45.3% overall. Most participants were female (87.4%) and worked as assistant nurses (73.1%). The average age was approximately 47 years. Participants were predominantly employed in the medium-sized municipality (52.2%) and had an average tenure of 17 years in eldercare.

Regarding providers, 54% were employed in public facilities, 17% in outsourced facilities, and 29% in private residential care homes. The outsourced sector had a notable portion of its workforce in part-time roles (64%), while 31% of the private sector employees had university degrees or equivalent, dropping to 19% when excluding registered nurses. This suggests a potential overqualification among basic-level staff, linked to broader observed trends,^58^ which show that foreign-born individuals often possess higher qualifications than required for their roles. Furthermore, many foreign-born workers – particularly those from outside Europe – are employed in roles such as care aides, positions that do not match their educational qualifications.^59^ Additionally, private sector workers had a lower average tenure at their current workplace. For more comprehensive information about provider types, see Table 1.

### Demands and resources compared between provider types

Domain-level JD-R variables showed no statistically significant differences, while there were five significant differences on a dimension level (**Q1**).

Emotional Demands differed significantly across groups (F(2, 250) = 3.42, p = .034) with a small effect size (eta-squared = .027). Tukey's HSD showed that outsourced employees (M = 68.3, SD = 18.1) experienced higher demands than those in public settings (M = 59.3, SD = 19.5), p = .025. Perceived influence differed significantly (F(2, 249) = 4.72, p = .010) with a small effect size (eta-squared = .036). Tukey's HSD indicated greater perceived influence in the private sector (M = 50.3, SD = 17.5) than in the public sector (M = 42.6, SD = 19.3), p = .013. Support from the supervisor differed significantly (F(2, 250) = 4.36, p = .014) with a small effect size (eta-squared = .034). Post-hoc analysis revealed higher supervisor support in the private sector (M = 75, SD = 24.6) than in the public sector (M = 65.5, SD = 28.4), p = .026. Conflict between work and personal life differed significantly (F(2, 249) = 3.69, p = .026) with a small effect size (eta-squared = .029). Tukey's HSD showed more conflict in the private sector (M = 67.1, SD = 23.1) than in the public sector (M = 56.2, SD = 31), p = .020. Leadership quality was statistically significant in the ANOVA (p = .042, eta-squared = .025). However, this significance did not persist in the post-hoc comparisons using Tukey's HSD. All other JD-R dimensions were non-significant in the ANOVA tests (see Supplementary Materials).

### Burnout

The mean burnout score was 43.69 (SD = 25.10), suggesting that most participants experienced symptoms of burnout part of the time. In the first GEE model (**1a**) focusing solely on provider type (**Q2**), a statistically significant difference in burnout was observed, with a higher score for burnout regarding outsourced workers than public care workers. In contrast, no statistically significant difference was found between private sector workers and public ones. When job demands, job resources, and covariates were accounted for in Model **1b** (**Q2**), the relationship for provider type was non-significant, although close (p = .054). Regarding municipalities, care workers in the medium-sized one reported statistically significantly higher burnout levels than those in the small one. Care aides also indicated statistically significant higher burnout levels than assistant nurses. As hypothesised, job demands were a statistically significant predictor of burnout, with increased job demands associated with higher self-rated burnout (**H1**) (Table 2). The results were non-significant for interaction effects between demands and resources and thus not included in the model (**H2**).

**Table 2.** Generalised estimation equation models for burnout among care workers across residential care home providers, accounting for job demands and resources and controlling for municipality, type of occupation, and gender.

Variables	Burnout					
	Model 1a			Model 1b		
Intercept	B (SE)	95% CI	Sig.	B (SE)	95% CI	Sig.
	41.42 (2.52)	36.48, 46.36	p < .001	32.53 (11.68)	9.64, 55.41	.005
Private provider	2.84 (3.38)	−3.79, 9.46	.402	0.57 (2.71)	−4.75, 5.88	.834
Outsourced provider	8.78 (3.19)	2.54, 15.03	**.006**	7.29 (3.77)	−0.11, 14.68	.054
Public provider (reference group)	0			0		
Small town				−8.80 (3.76)	−16.16, −1.43	**.019**
Large town				−3.77 (3.11)	−9.86, 2.33	.226
Medium-sized town (reference group)				0		
Women				2.35 (3.83)	−5.16, 9.85	.540
Men				0		
Other				−2.66 (5.53)	−13.50, 8.18	.630
Registered nurses				−2.01 (4.16)	−10.16, 6.14	.629
Care aides, education => 0–40 weeks				7.15 (2.22)	2.80, 11.49	**.001**
Assistant nurses (reference group)				0		
Demands at work				0.74 (0.14)	0.47, 1.00	**p < .001**
Work organisation and job content				−0.12 (0.13)	−0.38, 0.14	.374
Interpersonal relations and leadership				−0.22 (0.17)	−0.55, 0.12	.203
Social capital				−0.10 (0.14)	−0.38, 0.18	.487

Note: Bold figures indicate statistically significant results. Abbreviations: B = Beta Coefficient, SE = Standard Error, CI = Confidence Interval, Sig. = Statistical significance (p-value).

### Job satisfaction

For job satisfaction, the mean score was 65.84 (SD = 22.26), and about 60% of the respondents reported being satisfied or very satisfied with their work overall. In the first GEE model **2a**, care workers working for private providers reported higher job satisfaction scores than those working for public providers. In contrast, the result of outsourced vs public providers was non-significant (**Q2**). When adding JD-R variables, municipality, type of occupation, and gender in Model **2b**, the results of private provider vs public provider remained statistically significant (**Q2**). For the other variables in the model, lower job satisfaction scores were associated with being women, having higher tenure and job demands, and lower scores of interpersonal relations and leadership (**H1**). For the interaction effects, job demands interacting with work organisation and job content was statistically significant (**H2**) (Table 3).

**Table 3.** Generalised estimation equation models of job satisfaction among care workers across residential care home providers, accounting for job demands and resources and controlling for municipality, type of occupation, gender, age, and tenure.

	Job satisfaction					
Variables	Model 2a			Model 2b		
Intercept	B (SE)	95% CI	Sig.	B (SE)	95% CI	Sig.
	63.06 (2.75)	57.67, 68.45	p < .001	83.16 (22.88)	38.32, 128.00	p < .001
Private provider	7.27 (3.40)	0.62, 13.93	**.032**	5.71 (2.01)	1.77, 9.65	**.005**
Outsourced provider	3.61 (4.15)	−4.52, 11.73	.384	4.48 (3.39)	−2.17, 11.13	.187
Public provider (reference group)	0			0		
Small town				−2.64 (2.31)	−7.18, 1.89	.253
Large town				−2.21 (2.34)	−6.91, 1.94	.346
Medium-sized town (reference group)				0		
Women				−7.80 (2.15)	−12.01, −3.60	**p < .001**
Men				0		
Other				5.43 (11.20)	−16.53, 27.39	.628
Registered nurses				−0.37 (4.04)	−8.30, 7.55	.927
Care aides, education => 0–40 weeks				−0.02 (4.13)	−8.11, 8.08	.997
Assistant nurses (reference group)				0		
Age				−0.04 (0.12)	−0.28, 0.19	.713
Workplace tenure				−0.41 (0.18)	−0.76, −0.05	**.025**
Total tenure residential care homes				0.17 (0.11)	−0.05, 0.38	.136
Demands at work				−1.33 (0.36)	−2.03, −0.62	**p < .001**
Work organisation and job content				−0.57 (0.35)	−1.26, 0.11	.099
Interpersonal relations and leadership				0.69 (0.13)	0.44, 0.94	**p < .001**
Social capital				−0.07 (0.09)	−0.24, 0.10	.429
Job demands x Work organisation and job content				0.02 (0.01)	0.01, 0.03	**.005**

Note: Bold figures indicate statistically significant results. Abbreviations: B = Beta Coefficient, SE = Standard Error, CI = Confidence Interval, Sig. = Statistical significance (p-value).

### Turnover intention

The mean turnover intention score was 42.00 (SD = 27.64), and around 60% of respondents reported sometimes or more frequently considering leaving their job, while about 24% often or always considered leaving. There were no statistically significant differences between provider types in Model 3a (**Q2**). In Model **3b**, the turnover intention was statistically significant regarding provider type, with it being slightly lower among care workers in both outsourced and private homes than in public residential care homes (**Q2**). Job demands, interpersonal relations, and leadership were also statistically significant in the model (**H1**), and so was the interaction effect between job demands and work organisation and job content (**H2**) (Table 4).

**Table 4.** Generalised estimation equation models of turnover intentions among care workers across residential care home providers, accounting for job demands and resources and controlling for municipality and type of occupation.

	Turnover intention					
Variables	Model 3a			Model 3b		
Intercept	B (SE)	95% CI	Sig.	B (SE)	95% CI	Sig.
	44.26 (3.22)	37.95, 50.57	p < .001	26.74 (24.29)	20.87, 74.36	.271
Private provider	−5.90 (4.32)	−14.38, 2.57	.172	−3.99 (1.55)	−7.03, −0.94	**.010**
Outsourced provider	−3.19 (3.57)	−10.18, 3.80	.371	−5.20 (2.57)	−10.24, −0.16	**.043**
Public provider (reference group)	0			0		
Small town				−0.28 (2.12)	−4.44, 3.88	.895
Large town				−1.86 (2.17)	−6.10, 2.39	.390
Medium-sized town (reference group)				0		
Other				9.95 (13.18)	−15.89, 35.79	.450
Registered nurses				6.38 (4.74)	−2.91, 15.67	.178
Care aides, education => 0–40 weeks				−4.69 (3.87)	−12.26, 2.89	.225
Assistant nurses (reference group)				0		
Demands at work				1.52 (0.41)	0.72, 2.31	**p < .001**
Work organisation and job content				0.55 (0.37)	−0.19, 1.28	.143
Interpersonal relations and leadership				−0.69 (0.13)	−0.95, −0.43	**p < .001**
Social capital				0.08 (0.11)	−0.14, 0.31	.465
Job demands x Work organisation and job content				−0.02 (0.01)	−0.03, −0.004	**.009**

Note: Bold figures indicate statistically significant results. Abbreviations: B = Beta Coefficient, SE = Standard Error, CI = Confidence Interval, Sig. = Statistical significance (p-value).

## Discussion

This study examined differences in job demands and resources across public, outsourced, and private residential care home providers and associations between job demands, job resources, provider type, and the outcomes in terms of burnout, job satisfaction, and turnover intentions among care workers.

For Q1, we looked at differences in job demands and resources across provider types. We found a few significant differences. Emotional demands were significantly higher in outsourced settings than public ones, indicating a particular stressor within these environments. Perceived influence was rated higher in the private sector, suggesting a higher autonomy or control over work processes. Support from supervisors was also reported to be higher in the private sector. However, conflict between work and personal life was rated higher in the private sector, indicating one potential drawback of working in these settings.

For Q2, where the focus was on associations between provider types and care workers’ burnout, job satisfaction, and turnover intentions, our findings offer a mixed picture: outsourced workers reported higher levels of burnout compared to public care workers, but this difference was non-significant when job demands, job resources, and additional covariates were considered. In the comparative analysis, staff-rated emotional demands (Q1) were higher in outsourced than in public settings. Thus, when holding demands constant in the GEE models, this might explain why provider types now were non-significant for burnout. Job satisfaction was rated higher by private care workers than by public ones, indicating a difference in job satisfaction levels. However, care workers’ job satisfaction was above average in all provider types. The turnover intentions were slightly higher in public care compared to outsourced and private settings.

For H1, job demands were a significant predictor of burnout. This finding was consistent even when controlling for various covariates. However, the anticipated protective effect of job resources on burnout was not found, which is surprising considering the JD-R theory's hypothesis that job resources should buffer the negative impact of job demands on burnout.^32^ Job demands were associated with a decrease in job satisfaction. In contrast, interpersonal relations and leadership as a resource were associated with increased job satisfaction. Similarly, supportive social networks may be crucial in sustaining work retention among assistant nurses.^41^ Consequently, good leadership may be a necessary resource to consider in the JD-R theory. Higher job demands were associated with increased turnover intentions, which aligns with previous research.^3^ High demands may push care workers to consider leaving their positions, since turnover intention strongly predicts actual turnover.^60^ On the other hand, job resources such as interpersonal relations and leadership, in particular, seemed to mitigate these effects somewhat.

Regarding H2, our analysis revealed no significant interaction effects on burnout, indicating that the buffering role of job resources in the context of burnout might be less straightforward than hypothesised in the JD-R theory.^32^ Interestingly, in line with the theory, the interaction between job demands and work organisation and job content was statistically significant for job satisfaction, indicating that the relationship between job demands and job satisfaction may be moderated by how work is organised and how the job content is structured. Our analysis revealed a significant association between the interaction of job demands with work organisation and job content and the outcome of turnover intentions among care workers. This finding aligns with the JD-R theory's proposition that job resources can moderate the impact of job demands on employee outcomes.^32^ In this case, the structure and quality of work organisation and job content may influence how job demands influence care workers’ intentions to leave.

Our findings both support and differ from previous research. Szebehely et al.^3^ reported high job demands and turnover intentions in Swedish eldercare, with around 50% of care workers considering leaving their positions in residential care homes. In contrast, our study sample had a lower turnover tendency, with 60% sometimes considering to leave and 24% often or always considering to leave. This suggests that although job demands were higher regarding work pace and emotional demands, the turnover intentions are not as pronounced as expected based on previous findings. This could be due to municipal variation and different marketisation tendencies.^18,55^ Contrary to previous findings that indicated lower turnover intentions in nonprofit settings,^15^ our initial results showed no clear advantage for public providers. However, care workers at outsourced and private providers reported lower turnover intentions after adjusting for job demands and resources.

Regarding stress and its impact on health,^5,6^ our findings align with the general trend of substantial stress affecting care workers, who experience burnout symptoms part of the time. Duxbury et al.^4^ highlighted the impact of emotional strain on mental health for care workers, which is concerning given the higher emotional demands faced by care workers in outsourced settings. Outsourced workers reported slightly higher burnout levels, which may support previous observations of less favourable environments in outsourced settings.^13^ Care workers also tend to go beyond their duties to provide care,^61^ which could explain the high emotional demands.

Job satisfaction was generally high, with around 60% of the respondents satisfied or more than satisfied with their work. Aloisio et al.^26^ found no consistent relationship between job satisfaction and facility ownership. In contrast, our study identified higher job satisfaction in private settings compared to public ones. This may be linked to leadership and influence being rated higher among private providers than public ones, especially when considering that interpersonal relations and leadership were significant for job satisfaction. Previous research has also indicated that first-line managers at private providers have more autonomy over decision-making than their public sector counterparts,^43^ which could be relevant for being available to staff and, in turn, their job satisfaction.

Previous research^10^ and our study indicate mixed results regarding provider type and staff well-being. While we did not find any drastic differences in the psychosocial work environment based on provider type, more part-time staff in outsourced and private settings may hint at underlying differences in employment practices and priorities, possibly due to profit incentives, as Bos et al.^9^ discussed.

### Theoretical contributions and practical implications

Our findings support the JD-R theory, highlighting that elevated job demands are associated with increased burnout, higher turnover intentions, and decreased job satisfaction. This aligns with the health impairment process, which suggests that high job demands deplete employees’ mental and physical resources, leading to adverse outcomes such as burnout.^32^ Our findings also back up the motivational process proposed by the JD-R theory, demonstrating how job resources such as support from colleagues and managers, as well as good leadership, may create a supportive work environment, thereby improving care workers’ motivation and job satisfaction.^32^

Bakker and Demerouti^32^ recommended several aspects for further exploration concerning the JD-R theory, and our study addressed some of these. First, they suggest examining the buffering effect of job resources against job demands, which was a central component of our analysis. The partial support for the moderating role of resources suggests that further investigations are needed to examine how various demands and resources interact in diverse care environments. Second, our study also explored the JD-R theory in varied organisational settings, comparing public, outsourced, and private care homes. This aligns with the call for the theory to be further applied in diverse organisational settings. Care workers in outsourced settings reported higher emotional demands, and there was more reported conflict between work and personal life in the private sector compared to the public sector. This may indicate subtle differences in the psychosocial work environment across provider types.

Furthermore, the recent advancements in JD-R theory by Bakker et al.^42^ propose a multilevel approach where organisational factors influence managerial practices and staff outcomes. Our study supports this multilevel JD-R theory by demonstrating how the type of provider may have some influence that may affect job demands. Third, the role of leadership as an important job resource was also examined in our study. We assessed how leadership within these various settings impacts care workers. Our findings showed that supervisor support was higher in the private sector than in public care settings, which aligns with previous research.^43^ We also explored social capital as a job resource, in line with previous research.^38–40^ However, social capital was not significant as a resource for all outcomes in our study.

The findings from this study may have implications for policymakers and care sector stakeholders in Sweden and countries with similar welfare models or which utilise multiple provider types. We noted differences in the psychosocial work environment and well-being among different provider types, suggesting that market-oriented policies in eldercare might lead to differences in social care practices. This may reflect the challenges of meeting the goals of the Swedish Social Services Act,^62^ which calls for “equality in living conditions” in eldercare. It may also be related to the criticism of NPM principles.^20,22,23^ Variations in the work environment can lead to unequal care quality, as staff well-being is associated with the quality of care.^63,64^ Therefore, it is important to continually evaluate legislative goals and care policies supporting equitable care practices.

The noted differences between providers may indicate a need for targeted interventions that address the unique challenges in each setting, consistent with previous findings that provider type can influence the work environment of first-line managers.^43^ Public sector care workers reported lower job satisfaction and higher turnover intentions than private sector workers, who also rated supervisor support and autonomy higher, suggesting a need for better leadership in the public sector. Outsourced providers present different challenges, with care workers experiencing higher emotional demands, aligning with research that suggests that outsourced settings may impose additional burdens.^43^ Further investigation is needed to understand the causes of these demands in outsourced settings, which could inform the development of targeted interventions. Private care workers reported higher job satisfaction, possibly due to greater autonomy and supervisor support. However, the reliance on part-time staff in these settings can disrupt care continuity, undermining the relationship-based nature of care, which depends on stable staffing. Additionally, our findings indicate that conflicts between work and personal life were more common in private settings, where both outsourced and private providers had higher part-time staffing compared to the public sector. Private providers could focus on offering more stable, full-time positions and flexible working arrangements that better align with care workers’ personal time. Furthermore, Broms^26^ found that the pursuit of high profits in residential care homes may negatively impact staffing levels. Banerjee et al.^65^ advocate for stricter regulations, including capping profits in eldercare, to ensure that financial priorities do not compromise care quality and worker well-being. Policymakers should consider enforcing minimum staffing levels and capping profit margins to ensure that profit motives do not overlook the needs of care workers and users.

Despite the generally high level of job satisfaction reported, a significant number of care workers are considering leaving their jobs, echoing previous research.^3^ These findings highlight the need to improve the work environment across all types of care providers, both for the well-being of the care workers and to address the ongoing staffing challenges in eldercare. Improving the work environment is particularly important, considering that staff well-being is closely linked to the quality of care provided to care users.^63,64^ However, the persistent budget cuts in the sector make it increasingly difficult to achieve meaningful improvements, posing a significant challenge for the future of residential care.

### Strengths and limitations

The cross-sectional design limits the generalisability of the results. Although we controlled for various factors, such as type of occupation and municipality, the results may be more relevant for countries that structure their eldercare similar to the Nordic model. Nevertheless, the findings provide helpful information about three market-oriented municipalities that outsource care and involve private for-profit providers. Part of the findings may be relevant for other countries, as the marketisation of eldercare is not unique to Sweden.^1,2,22^

The study's sample size was limited, especially for the outsourced group. Considering that about one-third of the workforce in Swedish eldercare comes from non-European countries,^47^ it may be an oversight that we did not collect data on ethnicity. However, our sample was representative of gender, with the majority being women, which was in line with national levels.^46^ A strength of our research was that it accounted for clustering by residential care home, municipality and provider type in the analysis.

The response rate from registered nurses in our survey was notably low, especially among public providers. Swedish residential care homes on average employ 4.7 registered nurses for every 100 care recipients.^66^ In our study's 19 residential care homes, each typically had two to three registered nurses, amounting to approximately 35–40 across all facilities, but only 15 participated in the study. This low response rate could be due to the increasing use of consultant registered nurses. Private facilities generally employ on-site registered nurses, while public residential care homes rely more on consultant registered nurses.^67^ Given their non-permanent roles, these nurses are less likely to attend the workplace meetings where the survey was conducted, which potentially contributes to their underrepresentation in the survey responses. However, we controlled for occupation based on this and only found differences concerning care aides and assistant nurses.

Another limitation was that we did not measure more employment condition variables, which might have revealed more differences between for-profit and nonprofit providers. Rather, we focused on the psychosocial work environment and included questions concerning employment types. Our study's methodological approach, utilising COPSOQ III, allowed for a detailed assessment of job demands and resources, aligning well with the JD-R theory to evaluate the psychosocial work environment of care workers. Yet the comprehensive nature of COPSOQ, while extensively validated across various settings,^50^ might not have fitted the specific conditions of residential care homes that a more eldercare-focused survey could have. Despite this, its broad scope helped to thoroughly address possible job demands and resources, which is crucial for applying the JD-R theory.

## Conclusions

Our study contributes to understanding how job demands, resources, and the type of care home provider may influence care workers’ psychosocial work environment and well-being in residential care homes. We observed variations in the work environment across public, outsourced, and private residential care homes and found mixed results. We identified specific job demands, such as emotional demands, that were higher in outsourced settings than public ones, suggesting a more stressful environment in outsourced care homes. Conversely, job resources such as supervisor support were reported to be higher in private settings. These findings indicate a complex picture in evaluating differences in the psychosocial work environment, with subtle differences between providers. There is a particular need for better leadership and support in the public sector, addressing emotional demands in outsourced settings and encouraging full-time employment to support work-life balance in the private sector. Economic incentives in the care sector must not come at the cost of care workers’ well-being. Improving job resources across all provider types is necessary, but ongoing budget cuts make this difficult. Stakeholders must focus on improving job resources for care workers’ well-being amid financial pressures and profit-driven goals.

This study contributes to the JD-R theory by investigating both established and new elements, such as the impact of different organisational settings on job demands and resources. Practical implications from our findings suggest that reducing emotional demands or increasing resources to deal with them may be necessary in order to avoid burnout, especially in outsourced settings.

Considering the varied municipal contexts and potential differences, future research could benefit from a case study approach to explore the effects of market elements on care provision in depth. A comparative study of municipalities with and without market elements could provide valuable insights into whether differences between municipalities are more pronounced than those between provider types.

## Supplemental Material

sj-docx-1-wor-10.1177_10519815241300294 - Supplemental material for Does type of provider matter for staff well-being? a cross-sectional study of residential care home workers’ job demands and resources Supplemental material, sj-docx-1-wor-10.1177_10519815241300294 for Does type of provider matter for staff well-being? a cross-sectional study of residential care home workers’ job demands and resources by Tomas Lindmark, Sven Trygged and Maria Engström in WORK
