# Supplementary material for: Does type of provider matter for staff well-being? a cross-sectional study of residential care home workers’ job demands and resources
Source: Work. 2025 Jan 21;80(4):1822–37. doi: 10.1177/10519815241300294 (PMC12999991; doi:10.1177/10519815241300294)
Supplement: sj-docx-1-wor-10.1177_10519815241300294 - Supplemental material for Does type of provider matter for staff well-being? a cross-sectional study of residential care home workers’ job demands and resources [file sj-docx-1-wor-10.1177_10519815241300294.docx]

**Supplementary materials (Appendix)**

**Table 1A.** Descriptive statistics, Cronbach Alpha, and Pearson correlations for job demands, resources, demographic and outcome variables.

| Variable | 1 | 2 | 3 | 4 | 5 | 6 | 7 | M | SD | Alpha |
| --- | --- | --- | --- | --- | --- | --- | --- | --- | --- | --- |
| 1. Demands at work |  |  |  |  |  |  |  | 52.23 | 15.51 | .84 |
| 2. Work organisation and job content | -.15* |  |  |  |  |  |  | 65.26 | 14.24 | .77 |
| 3. Interpersonal relations and leadership | -.33*** | .59*** |  |  |  |  |  | 71.40 | 14.92 | .91 |
| 4. Social capital | -.33*** | .47*** | .76*** |  |  |  |  | 61.88 | 15.71 | .85 |
| 5. Age | .03 | -.17** | -.13* | -.10 |  |  |  | 46.9 | 11.50 | - |
| 6. Workplace tenure | .07 | -.10 | -.08 | -.07 | .32*** |  |  | 6.71 | 6.08 | - |
| 7. Total tenure residential care homes | .09 | -.11# | -.13* | -.14* | .59*** | .47*** |  | 16.97 | 11.79 | - |
| 8. Burnout | .53*** | -.25*** | -.35*** | -.33*** | -.05 | .03 | .04 | 43.69 | 25.10 | .85 |
| 9. Job satisfaction | -.35*** | .47*** | .62*** | .49*** | -.14* | -.17** | -.11# | 65.84 | 22.26 | - |
| 10. Turnover intention | .38*** | -.41*** | -.54*** | -.42*** | -.01 | .04 | .09 | 58 | 27.64 | - |

Note: *** p < .001, ** p < .01, * p < .05, # p < .10. All variables significant at the .10 level or lower are included in the Generalised Estimating Equations (GEE) analyses.

**Table 2A**. Descriptive statistics and inferential test results for burnout, job satisfaction, and turnover intention concerning gender differences.

| Outcome | Gender  M (SD) | Mean comparison (Gender) |
| --- | --- | --- |
| Burnout | Men(n=26)  34.94 (28.09)  Women(n=220)  44.70 (24.86) | t-test  t(244)=-1.87 p=**.063** |
| Job satisfaction | Men(n=26)  Mean rank 152.44  Women(n=220)  Mean rank 120.08 | Mann-Whitney U test  U(244)=2107.5.  p=**.019** |
| Turnover intention | Men (n=27) 36.11 (27.15)  Women(n=219)  42.81 (27.66) | t-test  t(244)=-1.19  p=.235 |

Note: Bold figures indicate statistically significant results. All variables significant at the .10 level or lower are included in the Generalised Estimating Equations (GEE) analyses.

**Table 3A.** Analysis of variance comparing the psychosocial work environment across different provider types, using the Copenhagen Psychosocial Questionnaire (COPSOQ) dimensions and domains.

| Variable | Type of provider, Mean (SD), N | df | F | Sig. |
| --- | --- | --- | --- | --- |
| Quantitative demands | Public: 38.79 (20.68) n = 135  Outsourced: 45.44 (19.67) n = 42  Private: 43.72 (17.83) n = 75 | 2(249) | 2.582 | .078 |
| Work pace | Public: 59.56 (21.04) n = 136  Outsourced: 63.69 (19.87) n = 42  Private: 60 (20.65) n = 75 | 2(250) | 0.654 | .521 |
| Emotional  demands | Public: 59.25 (19.47) n = 136  Outsourced: 68.35 (18.13) n = 42  Private: 61.33 (20.94) n = 75 | 2(250) | 3.422 | **.034** |
| Role  conflicts | Public: 45.55 (20.41) n = 134  Outsourced: 44.05 (19.93) n = 42  Private: 46.96 (17.55) n = 74 | 2(247) | 0.308 | .735 |
| Influence | Public: 42.58 (19.26) n = 135  Outsourced: 41.82 (18.44) n = 42  Private: 50.25 (17.48) n = 75 | 2(249) | 4.716 | **.010** |
| Development opportunities | Public: 67.71 (18.07) n = 136  Outsourced: 69.05 (18.06) n = 42  Private: 69.67 (18.46) n = 75 | 2(250) | 0.302 | .739 |
| Variation in work | Public: 61.48 (26.05) n = 135  Outsourced: 60.12 (24.10) n = 42  Private: 59.80 (23.34) n = 74 | 2(248) | 0.125 | .882 |
| Meaning in work | Public: 88.13 (16.79) n = 136  Outsourced: 85.24 (21.67) n = 42  Private: 85.67 (21.42) n = 75 | 2(250) | 0.595 | .553 |
| Predictability | Public: 66.27 (18.21) n = 135  Outsourced: 63.10 (16.09) n = 42  Private: 64.50 (19.06) n = 75 | 2(250) | 0.574 | 0564 |
| Recognition | Public: 60.48 (24.45) n = 135  Outsourced: 62.20 (20.57) n = 42  Private: 63.50 (23.59) n = 75 | 2(250) | 0.410 | .664 |
| Role clarity | Public: 84.31 (13.77) n = 136  Outsourced: 82.74 (11.55) n = 42  Private: 81.56 (15.40) n = 75 | 2(250) | 0.978 | .378 |
| Leadership quality | Public: 54.73 (27.69) n = 134  Outsourced: 65.28 (23.20) n = 42  Private: 60.67 (22.49) n = 75 | 2(248) | 3.206 | **.042** |
| Social support from  supervisor | Public: 65.53 (28.36) n = 136  Outsourced: 75.30 (23.67) n = 42  Private: 75.50 (24.61) n = 75 | 2(250) | 4.364 | **.014** |
| Social support from  colleagues | Public: 80.24 (18.73) n = 136  Outsourced: 80.36 (14.90) n = 42  Private: 83.33 (17.36) n = 75 | 2(250) | 0.788 | .456 |
| Social  community at work | Public: 68.95 (20.58) n = 136  Outsourced: 69.47 (19.81) n = 42  Private: 67.98 (20.92) n = 75 | 2(250) | 0.106 | .899 |
| Quality of work | Public: 60.39 (18.10) n = 136  Outsourced: 58.93 (16.17) n = 42  Private: 63.50 (17.16) n = 75 | 2(250) | 1.139 | .322 |
| Work-life conflict | Public: 56.17 (31.03) n = 135  Outsourced: 58.93 (25.86) n = 42  Private: 67.11 (23.12) n = 75 | 2(249) | 3.694 | **.026** |
| Horizontal trust | Public: 63.43 (20.49) n = 134  Outsourced: 63.10 (18.51) n = 42  Private: 61.49 (17.65) n = 74 | 2(247) | 0.247 | .782 |
| Vertical trust | Public: 64.97 (18.15) n = 135  Outsourced: 67.86 (15.35) n = 42  Private: 70.50 (15.62) n = 74 | 2(248) | 2.580 | .078 |
| Organisational  fairness | Public: 54.63 (21.01) n = 134  Outsourced: 57.74 (19.34) n = 42  Private: 56.53 (19.32) n = 74 | 2(247) | 0.461 | .631 |
| Job demands  (Domain) | Public: 50.85 (15.42) n = 136  Outsourced: 55.38 (16.32) n = 42  Private: 52.94 (15.12) n = 75 | 2(250) | 1.487 | .228 |
| Work organisation and job contents  (domain) | Public: 65.02 (13.79) n = 136  Outsourced: 64.06 (15.50) n = 42  Private: 66.37 (14.44) n = 75 | 2(250) | 0.396 | .674 |
| Interpersonal relations and leadership (domain) | Public: 70.21 (15.29) n = 136  Outsourced: 72.79 (12.94) n = 42  Private: 72.79 (15.27) n = 75 | 2(250) | 0.936 | .394 |
| Social  capital  (domain) | Public: 61.04 (16.74) n = 135  Outsourced: 62.90 (14.46) n = 42  Private: 62.84 (14.53) n = 74 | 2(248) | 0.414 | .661 |

Note: Bold figures indicate statistically significant results.

* Additional information regarding the participants' education types.

|  | Public sector | Outsourced | Private | Total |
| --- | --- | --- | --- | --- |
| **Type of education**  No education in healthcare or care services  care aide training 10-20 weeks  care aide training 40 weeks  assistant nurse training 40 weeks  Registered nurse education  Specialist nursing education  Social work education  Physiotherapist education  Other education | 13 (10%)  4 (3%)  5 (4%)  79 (58%)  3 (2%)  0  0  1 (1%)  16 (12%) | 2 (5%)  4 (10%)  0  32 (76%)  2 (5%)  1 (2%)  0  0  0 | 7 (9%)  0  1 (1%)  37 (49%)  12 (16%)  1 (1%)  2 (3%)  2 (3%)  9 (12%) | 22 (9%)  8 (3%)  6 (2%)  148 (59%)  17 (7%)  2 (1%)  2 (1%)  3 (1%)  25 (10%) |
